# Black Representation in the Primary Care Physician Workforce and Its Association With Population Life Expectancy and Mortality Rates in the US

**DOI:** 10.1001/jamanetworkopen.2023.6687

**Published:** 2023-04-14

**Authors:** John E. Snyder, Rachel D. Upton, Thomas C. Hassett, Hyunjung Lee, Zakia Nouri, Michael Dill

**Affiliations:** 1 Office of Planning, Analysis, and Evaluation, Health Resources and Services Administration, US Department of Health and Human Services, Rockville, Maryland; 2 Office of Health Equity, Health Resources and Services Administration, US Department of Health and Human Services, Rockville, Maryland; 3 Oak Ridge Institute for Science and Education, Oak Ridge, Tennessee; 4 Now with Surveillance and Health Equity Science, American Cancer Society, Atlanta, Georgia; 5 Workforce Studies, Association of American Medical Colleges, Washington, DC

## Abstract

**Importance:**

Studies have suggested that greater primary care physician (PCP) availability is associated with better population health and that a diverse health workforce can improve care experience measures. However, it is unclear whether greater Black representation within the PCP workforce is associated with improved health outcomes among Black individuals.

**Objective:**

To assess county-level Black PCP workforce representation and its association with mortality-related outcomes in the US.

**Design, Setting, and Participants:**

This cohort study evaluated the association of Black PCP workforce representation with survival outcomes at 3 time points (from January 1 to December 31 each in 2009, 2014, and 2019) for US counties. County-level representation was defined as the ratio of the proportion of PCPs who identifed as Black divided by the proportion of the population who identified as Black. Analyses focused on between- and within-county influences of Black PCP representation and treated Black PCP representation as a time-varying covariate. Analysis of between-county influences examined whether, on average, counties with increased Black representation exhibited improved survival outcomes. Analysis of within-county influences assessed whether counties with higher-than-usual Black PCP representation exhibited enhanced survival outcomes during a given year of heightened workforce diversity. Data analyses were performed on June 23, 2022.

**Main Outcomes and Measures:**

Using mixed-effects growth models, the impact of Black PCP representation on life expectancy and all-cause mortality for Black individuals and on mortality rate disparities between Black and White individuals was assessed.

**Results:**

A combined sample of 1618 US counties was identified based on whether at least 1 Black PCP operated within a county during 1 or more time points (2009, 2014, and 2019). Black PCPs operated in 1198 counties in 2009, 1260 counties in 2014, and 1308 counties in 2019—less than half of all 3142 Census-defined US counties as of 2014. Between-county influence results indicated that greater Black workforce representation was associated with higher life expectancy and was inversely associated with all-cause Black mortality and mortality rate disparities between Black and White individuals. In adjusted mixed-effects growth models, a 10% increase in Black PCP representation was associated with a higher life expectancy of 30.61 days (95% CI, 19.13-42.44 days).

**Conclusions and Relevance:**

The findings of this cohort study suggest that greater Black PCP workforce representation is associated with better population health measures for Black individuals, although there was a dearth of US counties with at least 1 Black PCP during each study time point. Investments to build a more representative PCP workforce nationally may be important for improving population health.

## Key Points

**Question:**

Is Black representation in the US primary care physician (PCP) workforce associated with population health outcomes?

**Findings:**

In this cohort study of survival outcomes for 1618 US counties, Black PCPs operated in less than half of all counties during each of 3 time points assessed (2009, 2014, and 2019). On average, every 10-percent increase in county-level Black PCP representation was associated with 31-day higher age-standardized life expectancy among Black individuals. Higher Black PCP representation levels were also associated with lower all-cause mortality rates among Black individuals and with reduced mortality rate disparities between Black and White individuals.

**Meaning:**

These findings suggest that greater representation of Black PCPs in the PCP workforce is associated with improved survival-related outcomes for Black individuals.

## Teaser

This cohort study used data from US counties to assess the association of Black representation in the primary care physician workforce with population life expectancy and mortality rates.

## Introduction

Various studies have shown correlations between higher primary care service availability and better population health outcomes.^1,2,3,4,5,6,7,8,9,10,11^ For example, Basu et al^1^ demonstrated that higher county-level primary care physician (PCP) supply is associated with increases in life expectancy and decreases in cardiovascular, cancer, and respiratory cause-specific mortality. Despite the established public health benefits for primary care, access to primary care services remains uneven across the nation, partly due to an insufficient number and uneven distribution of PCPs.^12^ However, PCP workforce shortfalls are just one facet of existing accessibility challenges. Patients in the health care safety net—that is, those with geographic, financial, insurance-related, linguistic, racism- or discrimination-related, and other barriers—experience disproportionate difficulties in accessing primary care and other essential health services.^13,14,15,16,17,18,19^ Life expectancy disparities between Black and White individuals have persisted for decades and have improved only modestly over time.^20,21,22^ While primary care availability appears to be important for everyone, some studies stratified by race suggest that there may be a more powerful inverse association between access and mortality for Black individuals.^2,6^

Prior work suggests that racial and ethnic minority PCPs provide a disproportionately large share of care nationally to racial and ethnic minority individuals, low-income and uninsured patients, and other historically underserved groups.^23,24^ Accordingly, building a more racially and ethnically diverse physician workforce has been cited as a means for expanding access to high-need specialties; providing more culturally competent care to racial, ethnic, and linguistic minority populations; offering patients greater choice for seeking care in line with their preferences; strengthening care quality; reducing health disparities; and better meeting the needs of the nation’s diverse populace.^23,24,25,26,27,28,29,30,31^ However, Black individuals and other racial and ethnic minority individuals have historically been underrepresented in the majority of health professions that require multiple years of advanced training, including medicine.^32,33,34^

Beyond the moral imperative to create equitable health career opportunities for all and to build a more diverse, representative physician workforce, the literature on the beneficial health outcomes from doing so primarily appears to focus on care access and utilization, patient adherence, physician communication, and patient experience of care measures.^35,36,37,38,39^ Evidence around any potential gains in clinical and public health outcomes from diversifying the physician workforce is more limited, and existing work in this area often focuses specifically on physician-patient racial concordance.^25,40,41,42^ As such, this investigation explores whether there is a county-level association between the degree of Black representation in the primary care workforce and key population health markers, including all-cause mortality rates, age-adjusted life expectancy, and all-cause excess mortality rates, a measure of health disparities between Black and White individuals.

## Methods

This cohort study was deemed exempt from institutional review board review under US Department of Health and Human Services regulations by the Alpha Independent Review Board. Informed consent was waived because it was not practicable to obtain consent from large numbers of physicians for a retrospective study. Data in the American Medical Association (AMA) Physician Masterfile are commonly used for research and other purposes, and physicians may opt out from their information being listed in this data set. The study followed the Strengthening the Reporting of Observational Studies in Epidemiology (STROBE) reporting guideline.

### Independent Variables

This investigation was modeled partly after work by Basu et al^1^ to assess how primary care accessibility relates to public health outcomes, but this study examined the influence of Black representation levels. Specifically, PCPs were defined as the number of non–federally employed physicians, excluding medical residents, actively practicing in the contiguous US, Alaska, and Hawaii in the outpatient setting in general practice, family medicine, general internal medicine, and general pediatrics. Practice information was acquired from the AMA Physician Masterfile for 3 years (January 1 to December 31 for 2009, 2014, and 2019), and physician race and ethnicity data were retrieved from the Association of American Medical Colleges (AAMC) databases, which compile self-reported information from multiple sources, as described previously.^33,43^ The AAMC race and ethnicity data are presented as a single variable with the following categories: American Indian or Alaska Native, Asian or Asian American, Black or African American, Hispanic or Latino (of any race), Native Hawaiian or other Pacific Islander, White, and other; the latter category includes individuals either identifying with more than 1 race and ethnicity descriptor and those with unknown or unclassifiable information on race and ethnicity. County-level population data on race and ethnicity were sourced from the 2009 to 2019 American Community Survey 5-year estimates.^44^ Black representation levels in the PCP workforce were measured using the following formula:
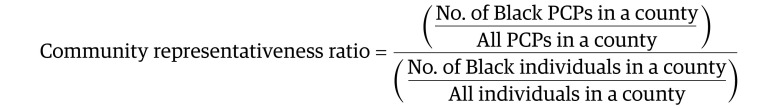
The community representativeness ratio is 1.0 when county-level Black representation levels in the PCP workforce match the proportion of community members identifying as Black. A representativeness ratio greater than or less than 1.0 indicates overrepresentation or underrepresentation of Black individuals in the PCP workforce relative to the community, respectively. This approach to measuring Black representation levels is advantageous because it is insensitive to both population and workforce magnitude, aiding in the comparison of counties of different size, and it is similar to the measure used in a recent publication looking at the racial and ethnic diversity of the health workforce.^45^ However, the ratio presented here uses a slightly different denominator—the whole population, rather than the working-age population—as the current study focused on population-level health care access instead of occupational opportunity. Both measures align with how the AAMC defines minority group underrepresentation in medicine and how this topic has been studied previously.^33,46,47^

### Outcomes

County-level, age-standardized life expectancy at birth and all-cause mortality rates (primary study outcomes) for 2009, 2014, and 2019 were derived from deidentified death records obtained through a data use agreement with the National Center for Health Statistics, using population counts from the US Census Bureau. Death records report race as a single variable (nonbridged), inclusive of Hispanic or Latino and non-Hispanic or non-Latino ethnicity, following 1997 guidelines from the Office of Management and Budget.^48^ Life expectancy and all-cause mortality rates were calculated for entire county populations and county Black populations using an approach aligned with the University of Wisconsin Population Health Institute^49^ County Health Rankings and Roadmaps program and using the equations presented in Arias et al.^50^ Life expectancy was defined as the estimated mean number of years a person could expect to live (from birth), according to age-specific mortality rates. A measure of the all-cause mortality rate disparity between Black and White individuals was also included as a study outcome variable. This disparity was calculated using the method applied by Benjamins et al^51^ to assess relative inequities between Black and White individuals using mortality rate ratios among these populations.

### Covariates

Following the example established by prior work, county-level covariates (Table 1) included the following: rural or urban designation,^53^ percentage living under the poverty threshold,^54^ percentage of uninsured individuals,^55^ median age,^56^ percentage who identified as Hispanic,^56^ ratio of men per 100 women,^56^ percentage with less than a high school degree,^57^ median home value,^58^ unemployment percentage,^59^ percentage of Medicare-enrolled individuals,^60^ age-adjusted percentage of adult tobacco smokers,^49^ percentage of adults with obesity,^49^ average daily density of fine particulate matter (air pollution),^49^ and number of hospital beds.^61^

**Table 1.** Characteristics and Covariates With Potential to Confound Measurement of the Association Between PCP Workforce Sufficiency and Life Expectancy or Mortality^a^

Characteristic	Mean (95% CI)			County change, 2009 to 2019^b^
	2009	2014	2019	
Independent variable				
Black PCP workforce ratio, median (95% CI)^b^^,^^c^	0.69 (0.63 to 0.74)	0.77 (0.71 to 0.84)	0.85 (0.80 to 0.92)	0.04 (0.03 to 0.06)
Total physicians per 100 000 population median (95% CI)^b^^,^^c^	61.90 (59.74 to 64.14)	61.11 (58.50 to 63.33)	60.19 (57.65 to 62.64)	0.14 (−0.52 to 0.48)
Covariate				
Age, y^d^	38.02 (37.81 to 38.23)	39.37 (39.14 to 39.60)	40.21 (39.97 to 40.44)	2.19 (2.09 to 2.28)
Hispanic population, %^d^	15.97 (15.97 to 15.98)	17.84 (17.84 to 17.85)	18.97 (18.96 to 18.97)	2.99 (2.98 to 3.00)
Sex ratio (men per 100 women), %^d^	96.33 (96.00 to 96.51)	96.54 (96.50 to 96.60)	96.67 (96.66 to 96.68)	0.34 (0.31 to 0.36)
Census rural county population, %^d^	8.51 (8.50 to 8.51)	8.18 (8.18 to 8.19)	8.01 (8.00 to 8.01)	−0.50 (−0.51 to −0.50)
Home value, $^d^	149 006.10 (144 107.10 to 153 905.10)	146 687.10 (142 525.10 to 150 849.2)	171 916.30 (166 643.80 to 177 188.70)	22 910.14 (21 469.96 to 24 350.31)
Poverty rate, %^c^	14.19 (14.18 to 14.19)	15.43 (15.43 to 15.44)	12.21 (12.20 to 12.21)	−1.98 (−1.98 to −1.97)
Uninsured rate for individuals aged <65 y, %^c^	17.22 (17.22 to 17.23)	13.51 (13.50 to 13.51)	10.77 (10.77 to 10.78)	−6.45 (−6.45 to −6.44)
Medicare enrollment, %^d^	NA^e^	NA	21.38 (21.15 to 21.62)	NA
Unemployed rate, %^d^	9.27 (9.27 to 9.28)	6.19 (6.19 to 6.20)	3.65 (3.65 to 3.66)	−5.62 (−5.63 to −5.62)
Less than high school education, %^d^	NA	NA	13.23 (12.96 to 13.50)	NA
Air pollution^d^	NA	NA	8.18 (8.11 to 8.26)	NA
Adult obesity, %^d^	NA	NA	33.83 (33.55 to 34.12)	NA
Adult smoking, %^d^	NA	NA	20.93 (20.73 to 21.13)	NA
No. of hospital beds, median (95% CI)^b^^,^^d^	NA	NA	121.50 (110.00 to 138.00)	NA
Dependent outcome variable				
Black life expectancy at birth, age-adjusted, y	76.64 (76.42 to 76.87)	77.06 (76.84 to 77.28)	77.12 (76.89 to 77.35)	1.09 (0.53 to 1.66)
Black age-adjusted mortality	906.19 (874.91 to 937.48)	880.21 (851.36 to 909.06)	868.33 (843.39 to 892.77)	−42.72 (−78.32 to −7.12)
Mortality rate disparity between Black and White individuals, median (range)^b^	1.13 (1.11 to 1.15)	1.11 (1.09 to 1.13)	1.10 (1.08 to 1.12)	−0.01 (−0.03 to 0.00)

Abbreviations: NA, not applicable; PCP, primary care physician.

^a^ All independent and dependent variables were reported at the county level for 1618 counties identified as having 1 or more Black PCPs. Counties that did not contain at least 1 Black PCP were not included. County-level age-adjusted life expectancy from birth estimates for Black individuals (based on the subsample of 1618 counties) were slightly higher than national estimates reported by the US Centers for Disease Control and Prevention National Vital Statistics System (NVSS).^52^ National life expectancy from birth estimates for Black individuals reported by NVSS was 74.50, 75.60, and 74.80 years for 2009, 2014, and 2019, respectively. The 95% CIs for the median were calculated using the CIQUANTDF option in SAS, version 9.4, to request nonparametric, distribution-free confidence limits for the 50th percentile (median). The 95% CIs for reported percentages (variables denoted with the “%” symbol) were calculated based on binomial proportion tests, which examined whether 2 proportions were equivalent or were statistically significantly different using 95% CIs. The number of physicians per county and the geographic variation in medical costs were both considered for inclusion as covariates but were ultimately excluded from the models because these variables were found to be highly collinear with the number of hospital beds.

^b^ Variables where the median is reported instead of the mean as a result of high levels of right skewness. In the analysis, right skewness was addressed through the log-transformation of these variables.

^c^ Indicates whether an estimator or study covariate was included in the final mixed-effects regression models as a time-varying covariate using data values from 2009, 2014, and 2019, such that between- and within-county influences for the estimator or study covariate were examined.

^d^ Indicates whether a study covariate was included in the final mixed-effects regression models for only 2019.

^e^ Cells with NA denote where values were not readily present in 2009 or 2014 for publicly available data sets.

### Statistical Analysis

This longitudinal analysis examined whether between- and within-county influences of Black PCP representation (as a time-varying covariate) were associated with county-level life expectancy and age-adjusted all-cause mortality rates for Black individuals, after controlling for covariates.^1^ Because Basu et al^1^ found that alternative geographic levels of study such as primary care service area and hospital referral region showed similar health care–seeking patterns, this study focused solely on county-level analyses. The combined sample comprised 1618 counties identified as having at least 1 Black PCP during 1 or more study time points (ie, 2009, 2014, or 2019) to ensure the use of nonzero representativeness ratios.

After testing several models for the level 1 residuals (eg, homoscedastic, autoregressive error structure, etc), mixed-effects growth models with an unstructured residual covariance matrix were used (1) to regress life-expectancy, age-adjusted all-cause mortality rates, and a log-transformed measure of mortality rate disparity between Black and White individuals on the log-transformed representativeness ratio within each county and (2) to estimate the between- and within-county components of variation for these outcomes, treating the Black representativeness ratio as a time-varying covariate.^62^ The outcome of all-cause mortality rate disparity between Black and White individuals and the aforementioned Black representativeness ratio were log-transformed to reduce positive skewness. To examine whether the associations between Black PCP representation and health outcomes were contingent on county poverty levels as a social determinant of health, moderation analysis assessed the statistical interaction of Black PCP representation with poverty. The association between the total number of PCPs per 100 000 population and each survival outcome also was tested to determine whether differences arose when comparing results for Black PCP representation vs all PCPs. *P* values were 2 sided, with α set to .05 to determine statistical significance. Sensitivity analyses and diagnostics examined (1) whether results met model-based assumptions and (2) if findings remained consistent when extreme residual observations or outliers were removed or when assessing the alternate representation ratio with the corrective constant. All analyses were performed in SAS, version 9.4 (SAS Institute). Data analyses were performed on June 23, 2022.

## Results

### Black Representation in the PCP Workforce

**Figure 1.**
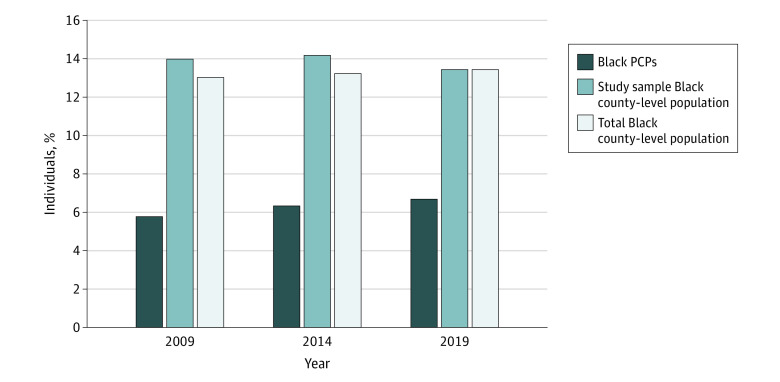
Percentage of Black Primary Care Physicians (PCPs) vs County-Level Black Population Across all study years, individuals identifying as Black comprised a smaller percentage of the PCP workforce relative to the study population of 1618 US counties and all 3142 US counties. The total Black county-level population percentage included all counties in the contiguous US, Alaska, and Hawaii.^63^

In this cohort study, Black PCPs comprised 6.3% of the combined sample (present in 1618 counties), and most counties (55.8%) with at least 1 Black PCP were urban. Median representativeness ratios (95% CIs) ranged from 0.69 (0.63 to 0.74) in 2009 to 0.85 (0.80 to 0.92) in 2019, suggesting that Black PCPs tended to be underrepresented relative to the county-level Black population. The percentage of Black PCPs for each time point was 5.7%, 6.3%, and 6.7%, respectively, whereas Black individuals comprised between 13.0% and 13.4% of the total US county-level population from 2009 to 2019 (Figure 1). In examining each time point, the number of counties with at least 1 Black physician was 1198, 1260, and 1308 counties in 2009, 2014, and 2019, respectively—consistently less than half of all 3142 Census-defined US counties as of 2014. There was a 9.8% increase in the number of US counties with 1 or more Black PCPs across this period. The percentage of US counties with 1 or more physician (irrespective of race and ethnicity) ranged from 90.9% to 94.2% in 2009, 2014, and 2019.

### Association of Black PCP Workforce Representation With Life Expectancy and All-Cause Mortality

Small improvements in age-adjusted life expectancy and mortality rates were seen nationally for Black individuals and for the US population between 2009, 2014, and 2019. Analyses were limited to the subset of 1618 counties with at least 1 Black PCP to ensure the use of nonzero representativeness ratios. In mixed-effects growth models, between-county influences of Black PCP representation indicated that a 10% increase in Black representation levels was associated with higher life expectancy for Black individuals by 30.61 days (95% CI, 19.13 to 42.44 days) ([0.88 × log(1.10)] × 365 = 30.61 days; statistical guidelines were used for interpreting log-transformed estimators in general linear and/or linear mixed models, while multiplying the final value by 365 to convert life expectancy to days based on a standard, 365-day calendar year^64^) and lower all-cause mortality among Black individuals by 12.71 deaths per 100 000 (95% CI, −14.77 to −10.66) (Table 2). A 10% higher level of Black representation in the PCP workforce also was associated with an estimated 1.2% lower disparity between Black and White all-cause mortality rates (95% CI, −1.29% to −1.05%), meaning that higher Black representation was associated with smaller mortality differences between Black and White individuals. Additionally, within-county influences suggested that during a given year of heightened workforce diversity, counties with higher-than-typical representativeness (relative to their average, underlying level of Black PCP representation) exhibited reduced mortality (−35.34 [95% CI, −58.86 to −11.81]) and a relatively smaller difference in all-cause mortality rates between Black and White individuals (−2.44 [95% CI, −3.65 to −1.23]).

**Table 2.** Results of Mixed-Effects Regression Models Associating Black PCP Representation and County-Level Covariates With Study Outcomes and Moderation Analysis^a^

Variable	Survival outcome for Black individuals			
	Model 1: life expectancy, y (95% CI)	Model 2: all-cause mortality rate (95% CI)	Model 3: log(mortality rate disparity between Black and White individuals) (95% CI)	Model 4: statistical moderation, life expectancy, y (95% CI)
Log(Black PCP workforce ratio)				
Between counties	0.88 (0.55 to 1.22)	−133.37 (−154.93 to −111.82)	−12.19 (−13.43 to −10.95)	0.59 (0.39 to 0.79)
Within counties	0.04 (−0.21 to 0.30)	−35.34 (−58.86 to −11.81)	−2.44 (−3.65 to −1.23)	0.06 (−0.19 to 0.32)
Interaction of between-counties log(Black PCP workforce ratio) × Poverty rate	NA^b^	NA	NA	0.04 (0.01 to 0.07)
Poverty rate				
Between counties	−0.10 (−0.15 to −0.06)	1.30 (−3.04 to 5.63)	−0.14 (−0.41 to 0.12)	−0.09 (−0.13 to −0.04)
Within counties	0.00 (−0.07 to 0.06)	2.25 (−5.10 to 9.59)	0.16 (−0.20 to 0.52)	−0.00 (−0.06 to 0.06)
Uninsured rate				
Between counties	−0.06 (−0.11 to −0.01)	1.55 (−3.36 to 6.46)	0.14 (−0.16 to 0.44)	−0.06 (−0.11 to −0.01)
Within counties	−0.14 (−0.23 to −0.06)	0.35 (−9.45 to 10.15)	0.05 (−0.42 to 0.52)	−0.14 (−0.22 to −0.05)
Time^c^	−0.23 (−0.52 to 0.06)	−17.57 (−51.43 to 16.29)	−0.03 (−1.65 to 1.59)	−0.22 (−0.51 to 0.07)
Ratio of men per 100 women, %	0.04 (0.02 to 0.06)	−3.88 (−5.65 to −2.10)	−0.21 (−0.32 to −0.11)	0.04 (0.02 to 0.06)
Rural status	1.06 (0.63 to 1.50)	−24.10 (−65.56 to 17.35)	−1.83 (−4.36 to 0.70)	1.06 (0.63 to 1.49)
Home value, $, median (95% CI)	0.02 (−0.00 to 0.04)	0.56 (−1.58 to 2.70)	0.20 (0.07 to 0.34)	0.02 (−0.00 to 0.04)
Hispanic population, %	0.04 (0.02 to 0.06)	−1.12 (−3.26 to 1.02)	−0.02 (−0.15 to 0.11)	0.03 (0.01 to 0.06)
Unemployed rate, %	0.11 (−0.05 to 0.26)	2.01 (−12.67 to 16.68)	0.73 (−0.17 to 1.62)	0.10 (−0.05 to 0.25)
Less than high school education, %	0.03 (−0.03 to 0.09)	0.02 (−5.86 to 5.90)	−0.09 (−0.45 to 0.27)	0.02 (−0.04 to 0.09)
Adult obesity, %	−0.08 (−0.12 to −0.04)	−0.80 (−4.54 to 2.94)	−0.20 (−0.42 to 0.03)	−0.07 (−0.11 to −0.03)
Adult smoking, %	−0.11 (−0.20 to −0.03)	21.45 (12.95 to 29.95)	0.57 (0.05 to 1.09)	−0.13 (−0.22 to −0.04)
Medicare enrollment, %	−0.02 (−0.06 to 0.02)	0.00 (−4.01 to 4.01)	−0.08 (−0.32 to 0.17)	−0.02 (−0.06 to 0.02)
Air pollution	−0.13 (−0.24 to −0.01)	20.74 (9.31 to 32.16)	1.27 (0.57 to 1.98)	−0.13 (−0.24 to −0.01)
Log(No. of hospital beds)	−2.65 (−0.95 to 4.35)	−95.62 (−268.90 to 77.66)	−1.20 (−12.10 to 9.71)	−2.46 (−0.75 to 4.16)

Abbreviations: NA, not applicable; PCP, primary care physician.

^a^ Median age was not included as a final study covariate due to issues regarding collinearity and a zero-order Pearson correlation between median age and Medicare enrollment percentage that exceeded 0.85. Unstandardized fixed effects with corresponding 95% CIs for between-county influences reported in Table 2 will neither match previously reported results of a 30.61-day increase in life expectancy, nor match between-county influence results for specified reductions in all-cause mortality and mortality rate disparities between Black and White individuals associated with a 10% increase in the (log-transformed) Black PCP workforce ratio.

^b^ Cells with NA denote cases where mixed-effects regression models did not include tests for statistical moderation, such that the interaction between the Black PCP workforce representativeness ratio and county-level poverty rates was not examined.

^c^ Time refers to a constructed variable generated such that 2009 was coded as 0, 2014 was coded as 1, and 2019 was coded as 2. This variable was used to assess whether each survival outcome exhibited statistically significant increases or decreases over time.

### Moderation Analysis

**Figure 2.**
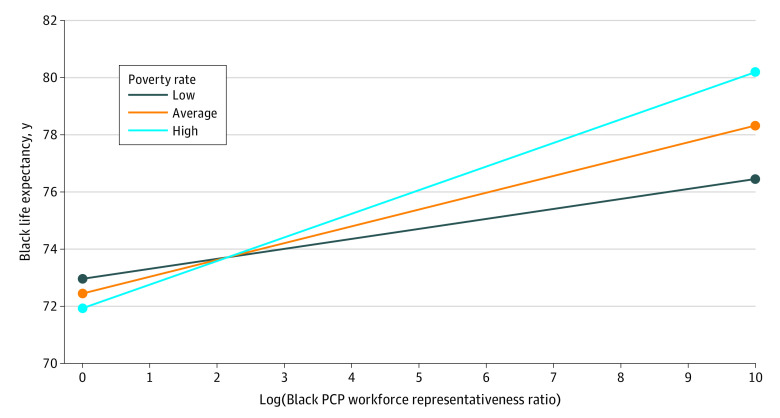
Statistical Moderation Analysis: Plot of 2-Way Interaction Between the Log-Transformed Black Primary Care Physician (PCP) Workforce Representativeness Ratio (Between-County Influence) With Poverty Rates (Between-County Influence) Estimated values (95% CIs) for the simple slopes at low poverty rates (1 SD below the mean), average poverty rates (0 for mean-centered poverty), and high poverty rates (1 SD above the mean) were 0.34 (0.07 to 0.62), 0.59 (0.39 to 0.79), and 0.83 (0.58 to 1.09), respectively. The simple slopes depicting the association between Black PCP representation and life expectancy were statistically significant at each level of poverty (low, average, and high), yet were greater for counties with high poverty (ie, with the simple slope for high poverty being equal to 0.83) compared with those with low or average poverty levels.^65^ Further, in terms of percentage increases, these results can be mathematically reformulated to show that a 10% increase in Black PCP workforce representation is associated with an 11.83-day (95% CI, 2.44 to 21.57), 20.53-day (95% CI, 13.57 to 27.48), or 28.87-day (95% CI, 20.18 to 37.92) increase in life expectancy for low, average, and high poverty levels, respectively. This mathematical reformulation is based on standard statistical guidelines for interpreting log-transformed predictors in general linear or linear mixed models.^64^

In examining the statistical interaction between Black PCP representation and poverty (0.04 [95% CI, 0.01-0.07]; Table 2), increases stemming from the between-county influence of Black physician representation were associated with enhanced life expectancy among Black individuals across all levels of poverty. Yet enhanced life expectancy was greater among US counties with high poverty, relative to counties with low to average poverty (Figure 2).

### Association Between Total PCPs and Outcome Measures

Mixed-effects growth models indicated that after controlling for study covariates, only the within-county influence of the total number of PCPs per 100 000 population was inversely associated with disparities in all-cause mortality rates between Black and White individuals (−1.16 [95% CI, −2.04 to −0.28]; Table 3).

**Table 3.** Results of Mixed-Effects Regression Models Associating Total Primary Care Physicians per 100 000 Population and County-Level Covariates With Study Outcomes^a^

Variable	Survival outcomes for Black individuals, model 5: log(mortality rate disparity between Black and White individuals) (95% CI)
Log(total PCPs per 100 000 population)	
Between counties	0.68 (−0.89 to 2.24)
Within counties	−1.16 (−2.04 to −0.28)
Poverty rate	
Between counties	0.35 (0.01 to 0.70)
Within counties	0.32 (−0.33 to 0.97)
Uninsured rate	
Between counties	0.56 (0.20 to 0.92)
Within counties	0.05 (−0.66 to 0.75)
Time^b^	0.51 (−2.07 to 3.08)
Ratio of men per 100 women, %	−0.22 (−0.34 to −0.10)
Rural status	−14.43 (−17.51 to −11.36)
Home value, $, median (95% CI)	0.72 (0.54 to 0.89)
Hispanic population, %	−0.16 (−0.32 to −0.01)
Unemployed rate, %	0.41 (−0.73 to 1.54)
Less than high school education, %	0.08 (−0.32 to 0.48)
Adult obesity, %	0.46 (0.20 to 0.72)
Adult smoking, %	0.28 (−0.33 to 0.89)
Medicare enrollment, %	−0.19 (−0.49 to 0.11)
Air pollution	7.17 (6.33 to 8.00)
Log(No. of hospital beds)	8.96 (−9.09 to 27.01)

Abbreviation: PCP, primary care physician.

^a^ Median age was not included as a final study covariate due to issues regarding collinearity and a zero-order Pearson correlation between median age and Medicare enrollment percentage that exceeded 0.85.

^b^ Time refers to a constructed variable generated such that 2009 was coded as 0, 2014 was coded as 1, and 2019 was coded as 2. This variable was used to assess whether each survival outcome exhibited statistically significant increases or decreases over time.

## Discussion

In this cohort study, moderate workforce diversity gains occurred in the 10-year period from 2009 to 2019, with a 9.8% increase in the number of US counties with 1 or more Black PCPs. In 2019, US Census population estimates reported that more than 70% of all US counties (excluding Puerto Rico) had 1 or more Black residents; however, the results of this study suggested that over half of all US counties had no Black PCPs during each time point. Among the counties that did, Black PCPs tended to be underrepresented relative to the Black county-level population (ie, median representativeness ratios <1.00). Comparatively, the percentage of US counties with 1 or more PCP (irrespective of race and ethnicity) ranged from 90.9% to 94.2% in 2009, 2014, and 2019.

This longitudinal study used multilevel or mixed-effects growth models to examine counties with 1 or more Black PCPs to determine whether increases in Black PCP representation levels were associated with better mortality outcomes among Black individuals. Greater Black PCP representation levels were associated with longer life expectancy and were inversely associated with all-cause mortality rates for Black individuals. Greater representation also was associated with a smaller difference in all-cause mortality rates between Black and White individuals. Moderation analysis suggested that the association between Black PCP representation and life expectancy was greater in counties with high poverty levels compared with counties with low or average poverty levels. Primary care availability, as measured by the total number of PCPs per 100 000 population, did not have a statistically significant association with life expectancy or mortality rates among Black individuals after controlling for other covariates, while within-county influences were associated with a reduced difference in all-cause mortality rates between Black and White individuals. Taken together, these findings suggest that Black PCP workforce representation levels are relevant to and potentially affect Black population health.

This investigation builds on prior work demonstrating the importance of primary care as well as the value of diversity, inclusion, and equity in the PCP workforce. Primary care physicians are a source of continuous, comprehensive care for their patients, serving to prevent and manage disease across the lifespan and coordinating the care provided to their patients elsewhere in the health care system. In addition, PCPs promote patient physical, mental, and general health and well-being; engage patients in actively participating in the management of their own health; often address the broader determinants of health within patients’ environment; and work to ensure equitable patient access to necessary health resources.^66,67^ Various studies have shown correlations between the higher availability of primary care services and desired population health outcomes, such as lower all-cause and cause-specific mortality.^1,2,3,4,5,6,7,8,9,10,11^ Racial differences frequently observed in population health outcomes studies are generally considered to result from fixable health system factors, such as differences in the availability and quality of care.^68,69,70^ Race as a study variable, in and of itself, is not considered a biological determinant of health outcomes; rather, it is a social construct that serves as a proxy measure for the structural inequities inherent in our society, and specifically in the health system.^69,70^ Empirical evidence shows that individuals belonging to minority racial and ethnic groups experience discrimination within the US health care system that adversely affects their access to, utilization of, experience in receiving, and outcomes from health care services.^17,19,71,72,73^

Physician-patient race concordance for Black individuals appears to often be associated with improved outcome metrics in some of these arenas, and some Black patients may prefer to seek care from racially concordant physicians due in part to the value placed on certain shared aspects of culture and experience.^23,35,37,38,39,74,75^ Although building a more diverse and representative physician workforce should not be a means to reinforce care segregation or to deemphasize the need to strengthen all physicians’ cultural competency, it does broaden patients’ choices for selecting PCPs and may offer outcome benefits for Black and other racial and ethnic minority patients.^35,37,38,39,42^ A more diverse workforce in research and leadership roles, able to leverage a wide array of personal and professional experiences in such positions, can additionally aid in shaping more broadly relevant and inclusive research and policy agendas.^28^

However, Black individuals are underrepresented in the majority of health professions that require multiple years of advanced training, including medicine, and numerous barriers limit the entry of Black students into medical careers.^32,33,34,76,77,78^ Potential interventions to address this include implementing changes in the processes for admissions, hiring, and promotions at universities, such as holistic review, and efforts to better nurture an educational and training environment that is structured for inclusion.^79,80^ The Health Resources and Services Administration, the primary federal agency supporting health care delivery to geographically isolated and medically underserved individuals, offers resources to support building an increasingly diverse national health workforce through its Health Careers Opportunity Program, Scholarships for Disadvantaged Students program, and Centers of Excellence program.^32^ Efforts to expand structural diversity within the health workforce, meaning to improve the numeric or proportional racial and ethnic mix of practicing PCPs, can be complemented by other strategies. These include strengthening cultural competency curricula and implementing educational approaches that elevate the principles of diversity, equity, and inclusion, such as engaging health professional students and trainees in diverse learning experiences in terms of race and ethnicity and gender (curricular diversity) and providing opportunities for students and trainees to interact with peers from different racial and ethnic backgrounds than themselves (interactional diversity).^81,82^ Example programs include Doctors Back to School from the AMA^83^ and the Action Collaborative for Black Men in Medicine from the AAMC,^84^ in partnership with the National Medical Association.

### Limitations

This study has several limitations. Although Basu et al^1^ found that health care–seeking patterns were similar across different levels of geography, this study was performed solely at the county level, and people do not necessarily seek primary care solely in their county of residence. Further, geographic proximity to health care is not equivalent to access. Since race and ethnicity was captured as a single variable in the PCP data set used for this analysis, this means that only physicians who self-identified as Black were characterized as such. In addition, life expectancy and mortality are multifactorial concepts, and mortality data categorized using race and ethnicity–based markers do not describe homogenous populations. This study attempted to control for important covariates with potential to confound the results (eg, health insurance access) but additional cultural factors likely play a role, including language and immigration status, although these are difficult to account for with currently available data. The associations identified between Black representation and the study outcomes do not imply causation. This study also does not investigate whether physician-patient racial concordance is occurring during care delivery. County-level Black representation in the physician workforce may serve as a marker for other community-based and health system factors that affect living environments and health outcomes for Black individuals.

## Conclusions

In this longitudinal cohort study of the PCP workforce in US counties where there were Black PCPs, higher levels of Black representation within the physician workforce were observed to be directly associated with longer life expectancy and inversely associated with all-cause mortality rates and all-cause mortality rate disparities for Black individuals. Hence, Black representation levels likely have relevance for population health, supporting the need to expand the structural diversity of the health workforce. Future investigations may examine the likely myriad factors behind this finding, the extent to which physician-patient racial concordance plays a role in this observation, and the effects that efforts to diversify the health workforce ultimately have on population health.

## Notes

Supplement.
Data Sharing Statement
