# Supplementary material for: Black Representation in the Primary Care Physician Workforce and Its Association With Population Life Expectancy and Mortality Rates in the US
Source: JAMA Netw Open. 2023 Apr 14;6(4):e236687. doi: 10.1001/jamanetworkopen.2023.6687 (PMC10105312; doi:10.1001/jamanetworkopen.2023.6687)
Supplement: Supplement. — Data Sharing Statement [file jamanetwopen-e236687-s001.pdf]

Data Sharing Statement

Snyder. Black Representation in the Primary Care Physician Workforce and Its Association With Population Life Expectancy and Mortality Rates in the US. *JAMA Netw Open*. Published April 14, 2023. doi:10.1001/jamanetworkopen.2023.6687

Data
Data available: No

Additional Information
Explanation for why data not available: The physician race and ethnicity data used in this project were retrieved from the Association of American Medical Colleges (AAMC) databases and are proprietary. As such they cannot be publicly shared without first establishing a Data Use Agreement with the AAMC.
